# Community-based wound management in a rural setting of Côte d’Ivoire

**DOI:** 10.1371/journal.pntd.0010730

**Published:** 2022-10-13

**Authors:** Simone Toppino, Didier Yao Koffi, Bognan Valentin Kone, Raymond T. A. S. N’Krumah, Ismaël Dognimin Coulibaly, Frank Tobian, Gerd Pluschke, Marija Stojkovic, Bassirou Bonfoh, Thomas Junghanss

**Affiliations:** 1 Division Infectious Diseases and Tropical Medicine, Heidelberg University Hospital, Heidelberg, Germany; 2 Centre Suisse de Recherches Scientifiques en Côte d’Ivoire, Abidjan, Côte d’Ivoire; 3 Programme National de Lutte contre l’Ulcère de Buruli, Abidjan, Côte d’Ivoire; 4 Université Félix Houphouët-Boigny d’Abidjan, Abidjan, Côte d’Ivoire; 5 Université Peleforo Gon Coulibaly de Korhogo, Korhogo, Côte d’Ivoire; 6 Molecular Immunology Unit, Swiss Tropical and Public Health Institute, Basel, Switzerland; 7 University of Basel, Basel, Switzerland; Minia University, EGYPT

## Abstract

**Background:**

Wounds are a neglected health problem in rural communities of low-income countries, mostly caused by trauma and ulcerative skin diseases including Neglected Tropical Diseases (NTDs) and associated with systemic complications and disability. Rural communities have limited access to high quality health services-based wound care.

**Methods:**

We conducted a prospective observational study on wound management at three levels–community (C), health centre (HC), district hospital (DH)—in a rural community of Côte d’Ivoire. Patients with skin wounds actively identified in a house-to-house survey and passively in the health services in a defined area of the Taabo Health and Demographic Surveillance System were asked to participate and followed-up longitudinally. Endpoints were proportion of wounds closed, time to wound closure, wound size over time, frequency of secondary bacterial infection, need for recapturing after follow-up interruption, and duration of treatment stratified by health service level and wound aetiology.

**Results:**

We enrolled 561 patients with 923 wounds between May 2019 and March 2020. The observation period ended in March 2021. Median age was 10 years (IQR 7–15), 63.0% of patients were male. Almost all (99.5%, 870/874) wounds closed within the observation period, 5.3% (49/923) were lost to follow-up. Wounds primarily treated in C, HC and DH closed within a median time of 10, 16 and 170 days, respectively. Median time to acute wound and chronic wound closure was 13 and 72 days, respectively. Wounds treated in C, HC and DH presented with secondary bacterial infections in 10.3% (36/350), 31.0% (133/429) and 100% (5/5) of cases, respectively. Recapturing was required in 68.3% (630/923) of wounds with participants reporting wound closure as the main reason for not attending follow-up.

**Conclusions:**

We describe a wound management model based on national and WHO recommendations focusing on early identification and treatment in the community with potential for broad implementation in low-income countries.

**Trial registration:**

Registration at ClinicalTrials.gov (NCT03957447).

## Author summary

Wounds are a neglected health problem in rural communities of low-income countries, mostly caused by trauma and ulcerative skin diseases including Neglected Tropical Diseases (NTDs) and associated with systemic complications and disability. Rural communities have limited access to high quality health services-based wound care.

We conducted a prospective observational study on WHO recommendation-based wound management at three levels–community, health centre, district hospital—in a rural community of Côte d’Ivoire. The healthcare personnel were trained in wound identification and treatment. Patients with skin wounds of all causes identified actively in a house-to-house survey and passively in the health services in a defined area of a Health and Demographic Surveillance System were included in the study and followed-up longitudinally.

Most wounds could be treated with basic wound care in the community or at the health centre by local healthcare personnel and closed within 2 to 3 weeks. Few wounds required referral to the district hospital or specific treatment. The results of our study indicate that a community-centred wound management model based on WHO recommendations and focusing on early identification and treatment of all wounds independent of the cause has potential for broad implementation in low-income countries.

## Introduction

Wounds are a common health problem caused by everyday traumas (particularly affecting children), road traffic and occupational accidents, medical interventions and ulcerative skin and systemic diseases, including neglected tropical diseases (NTDs), such as Buruli ulcer (BU), yaws, lymphatic filariasis and leprosy [1].

Independent of the cause, if not properly diagnosed and treated, wounds lead to local and systemic complications. Local complications include chronic wound development, loss of tissue, loss of function due to scarring and contractures and in severe cases loss of limbs. Systemic complications include life threatening sepsis, rheumatic fever, and endocarditis. Wounds, particularly those caused by skin NTDs, have a significant negative socio-economic impact that derives from direct and indirect costs of wound care, stigmatization and social isolation [2]. Chronic wounds due to skin NTDs are also associated with mental health issues such as psychological distress, anxiety, depression and other mental health conditions [3,4].

Rural communities in low-income countries often resort to traditional treatments, which can be dangerous for large wounds and wounds in critical anatomical sites. In ulcerative skin diseases, such as Buruli ulcer, specific treatment is, in addition, withhold. Access to health services is limited due to distance and constrained resources, including training of healthcare personnel in wound management. The national control programs for neglected tropical diseases of the skin (skin NTDs) including BU and yaws, provide free drug treatment and wound care in reference centres, although this is often not fully implemented. Wounds outside these programs receive less attention [5,6].

Identified and treated early with adherence to a basic set of WHO recommendations wounds usually heal fast [6,7]. WHO promotes a new integrated strategy for skin NTDs targeting all wounds. Training packages for healthcare personnel, including community healthcare workers, and the establishment of referral pathways for early diagnosis and treatment are the main components of this strategy [8]. A study on decentralized community-based treatment of BU carried out in Benin has shown that treatment at the community level can be effective for non-severe cases and is well received by the local population [9]. Here, we report the results of a prospective observational study on wound management in Côte d’Ivoire with a focus on early identification and treatment in the community.

## Methods

### Ethics statement

The study protocol has been approved by the Comité National d’Éthique de la Recherche (CNER), Côte d’Ivoire (N° 081-18/MSHP/CNESVS-km) and the Ethical Review Board of Heidelberg University Hospital (N° S-797/2018). The study has been registered at ClinicalTrials.gov under the registration number NCT03957447. Written consent was obtained from all participants aged 18 years or older, and from parents, caretakers, or legal representatives of participants younger than 18 years.

### Study objective

The objective of the study was to describe a community-based wound management model focusing on early identification and treatment in a rural setting and to characterize its performance by measuring the proportion of wounds closed, time to wound closure, wound size over time, frequency of secondary bacterial infection, need for recapturing after follow-up interruption, and duration of treatment stratified by health service level and wound aetiology.

### Study setting, design, and participants

We conducted a prospective observational study on community-based wound management at three levels (community, health centre, district hospital) in the Ahondo Health Area, Tiassalé district, Côte d’Ivoire which is part of Taabo Health and Demographic Surveillance System (HDSS) [10–12]. The setting is described in detail in [13].

We carried out a cross-sectional study combining active (household-based survey) and passive case finding (health services-based survey) in the same area to determine the prevalence and clinical epidemiology of wounds in a rural community of Côte d’Ivoire [13].

**Fig 1:**
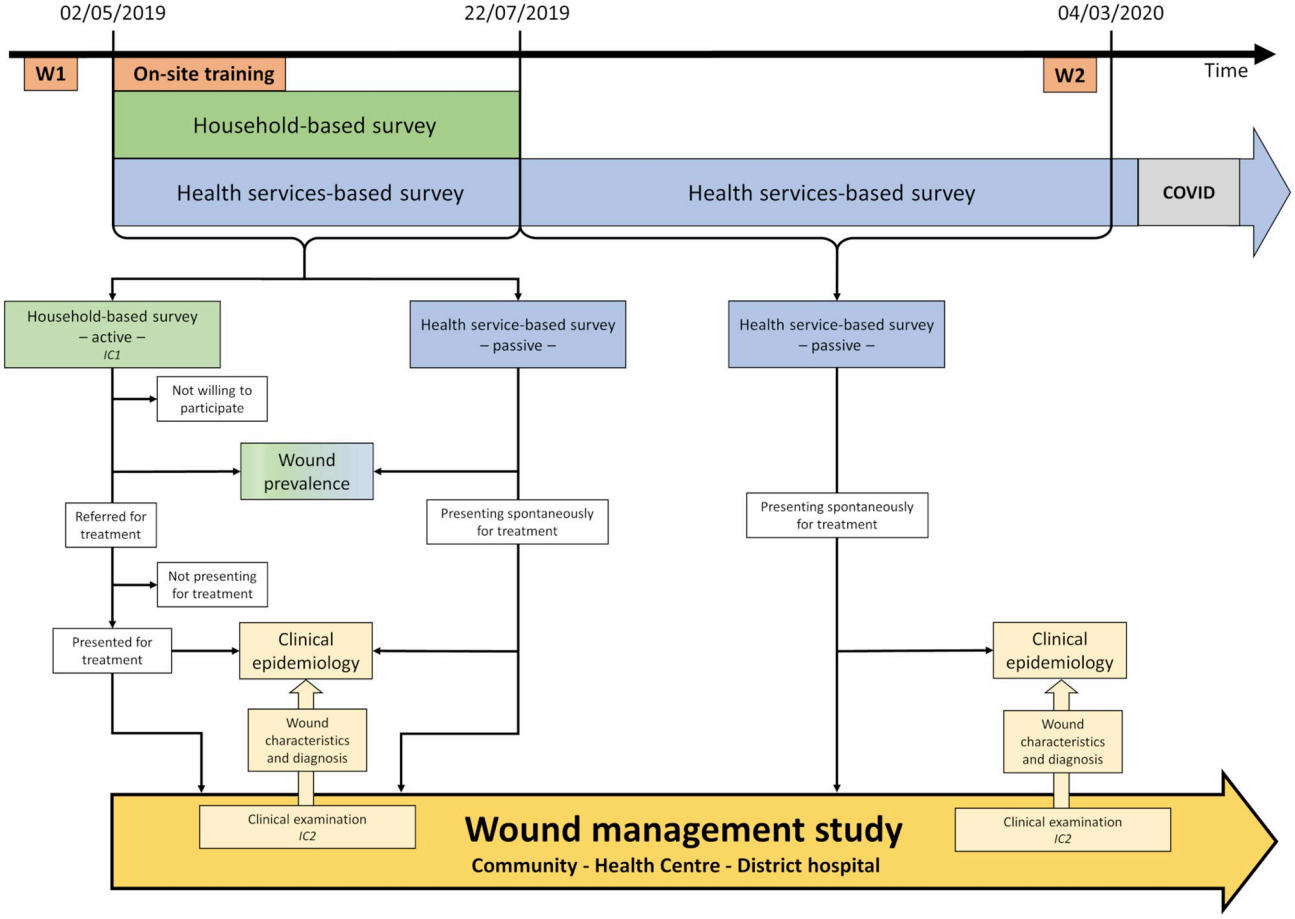
Study flow chart: interaction between household- and health services-based surveys and wound management study. Patients identified in the household- and health services-based surveys were offered to participate in the wound management study. The wound management study was carried out at three levels, in the community (community health workers—CHWs), health centre and district hospital according to the capability of the various levels and with the aim to identify and treat wounds as early as possible. The health staff of all three levels were trained in identifying, classifying, and treating wounds. W1 and W2: Training workshops (W) of nurses, assistant nurses, and CHWs; On-site training. IC1 and IC2: Informed consent (IC) for cross-sectional study and wound management study, respectively.

The interaction between the two studies is summarized in Fig 1.

Patients enrolled in the household- and health services-based survey were offered to participate in the wound management study. Enrolment took place in the community, at the Ahondo Health Centre (AHC) and at the wound management unit of Taabo district hospital (WMU). After enrolment patients were referred to the most appropriate health service based on the severity of the wound(s).

We define wound management as the diagnosis, treatment and follow-up of wounds including infrastructure, wound dressing material, a referral system, and psychosocial support based on WHO recommendations [3]. Local healthcare personnel were trained in wound management in two workshops, before the start of the study and in a refresher workshop, and received additional training on-site.

Independently of agreeing to participate in the study all patients were offered the same free care. There were no exclusion criteria.

At enrolment, the trained local healthcare personnel captured relevant patient information, examined the patient, including detailed assessment of the wound, made a presumptive clinical diagnosis and initiated treatment accordingly. Preferably, treatment was provided in the community and in more demanding cases in AHC or at the WMU. Patients were followed up until the wound had closed. Clinical data were collected at each visit. Data collected and diagnostic criteria are described in detail in [13]. A reduced data set was collected in the community by CHWs (simple wounds).

Establishing the presumptive diagnosis was the starting point of the wound management study. Presumptive diagnoses were regularly reviewed at follow-ups of the wounds. In case of treatment failure, the cause of treatment failure was analysed and it was decided if the current diagnosis could be maintained, but treatment had to be adapted / reinforced / intensified or both the diagnosis and treatment had to be revised [13].

### Wound management

Wound management was based on WHO [7,8] and national recommendations and, for specific aetiologies such as BU and yaws, additionally, on the national control programs.

Wounds with secondary bacterial infection, particularly with deep or systemic infection, and wounds requiring suturing or debridement were treated at AHC by assistant nurses supervised by a nurse. Wounds requiring intensive wound management or surgical treatment were treated at the WMU. Upon clinical improvement patients were referred and treated as close to their homes as possible.

Follow-up visits were scheduled according to clinical judgment. Wound assessment was performed at each visit to assess treatment effectiveness and wound healing progress. Treatment failure was assessed applying Flanagan’s criteria, i.e. wound area reduction < 30% within 3 weeks +/- 3 days of treatment initiation [14].

Patients not attending follow-up appointments and not contacting the local healthcare personnel within 7 days (2 days for wounds considered severe by the personnel) were entered in a recapture list and contacted by CHWs or HDSS enumerators. Patients with persisting wounds were invited to continue treatment at the local health services and financial and psychosocial support were offered if socioeconomic reasons, mental health issues or cultural barriers for non-attendance were identified.

Clinical case discussions were conducted at regular intervals at Ahondo Health Centre and the WMU of Taabo District Hospital for the entire local healthcare personnel to provide an opportunity for continuous interactive teaching across health care levels and to reinforce wound management practices based on WHO recommendations.

Weekly virtual clinical conferences were held between clinical investigators in the field and the clinical team at Heidelberg University Hospital.

### Clinical data collection

Wound assessment, presumptive clinical diagnoses and treatment were documented on case report forms at enrolment and at each follow-up visit until wound closure, loss to follow-up or end of the study period. Wounds were photo documented at each visit with a high-resolution camera.

On recapture, closed wounds and persisting wounds were documented. Patients with persisting wounds were encouraged, if required repeatedly, to continue treatment and wound progress was documented.

### Infrastructure, materials, and psychosocial support

The infrastructure of AHC and the WMU was assessed. The former did not meet the requirements for WHO-recommended wound management. The infrastructure of AHC was enhanced with drinking quality water supply [15], wound showers, grey water and waste disposal system, examination beds and a lighting system. CHWs were equipped with backpacks containing the basic materials for local wound care in the community. Wound dressing material was provided free of cost to all patients presenting for wound treatment. AHC and pharmacy of Taabo district hospital were regularly stocked with dressing material. Local healthcare personnel managed the material distribution and placed orders in a timely manner to avoid shortages. Each health service location was equipped by the project with a mobile phone with a prepaid plan for calls, SMS, and internet to support communication between the local healthcare personnel for patient referral and with the clinical investigators for clinical decisional support. Patients who could not afford supplementary diagnostics (e.g. X-rays, CT-scan) or treatment (e.g. analgesics, antibiotics, surgical interventions) were supported by the project. Psychosocial support was provided by the study sociologist and, under his supervision, by a master student in social sciences. Interviews, discussions, and focused group discussions were conducted. Patients in need of psychosocial support were identified based on referral from local healthcare personnel, low attendance rates or through individual assessment for hospitalized patients.

### Data management and analysis

The data were analysed for proportion of wounds closed, time to wound closure, wound size over time (Flanagan criteria) [14], frequency of secondary bacterial infection (local, deep, or systemic) [16], the proportion of cases in need of recapturing after follow-up interruption, and duration of treatment, defined as time elapsed between enrolment and the last wound dressing change performed by local healthcare personnel, stratified by health service level where wounds had been treated (community, AHC, WMU) and by wound type at enrolment and at final diagnosis.

Patients lost to follow-up were excluded from the analysis on wound closure. Only wounds with a gap of 7 days or less between the last wound dressing change and documented wound closure were included in the analysis on time to wound closure.

The data were double-entered into a RedCap database [17,18] and cleaned. Statistical analysis was performed with RStudio (R Core team, Version 1.4.1106).

## Results

### Patient characteristics

Between May 2^nd^, 2019, and March 4^th^, 2020, 561 patients were enrolled into the wound management study, 410 during combined active and passive case finding, 151 during passive case finding only [13]. The dataset was finalized on March 4^th^, 2021. At the end of the study period, 39 patients were lost to follow-up, 1 of which died before wound closure (see S1 Notes—section A).

**Fig 2:**
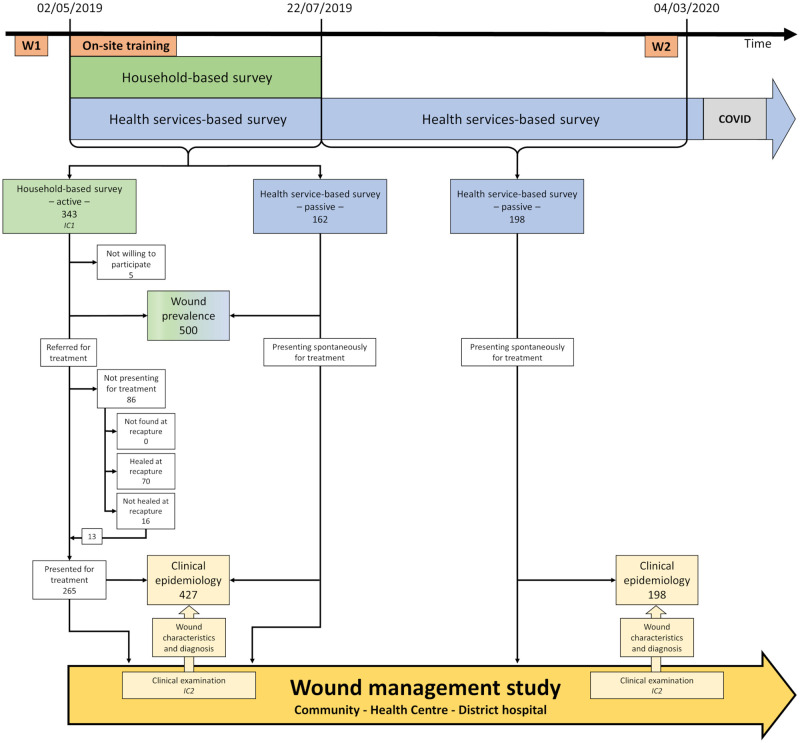
Enrolment of wound events into the wound management study during household- and health services-based surveys. Wound event was defined as an injury (e.g. mechanical trauma, burn, animal bite) or a specific pathology (e.g. BU, yaws) leading to one or multiple wounds. Wounds enrolled on the same date and attributed to the same aetiology were considered as one wound event. Specific aetiologies, such as BU or yaws, that could lead to multiple wounds over time were considered a single wound event. For details see [13].

Median age was 10 years, with 74.1% (403/544) of patients below the age of 15. 63.0% of patients were male, corresponding to a male to female ratio of 1.7. Fig 2 shows the distribution of wound events enrolled during the study period.

### Wound characteristics

**Fig 3:**
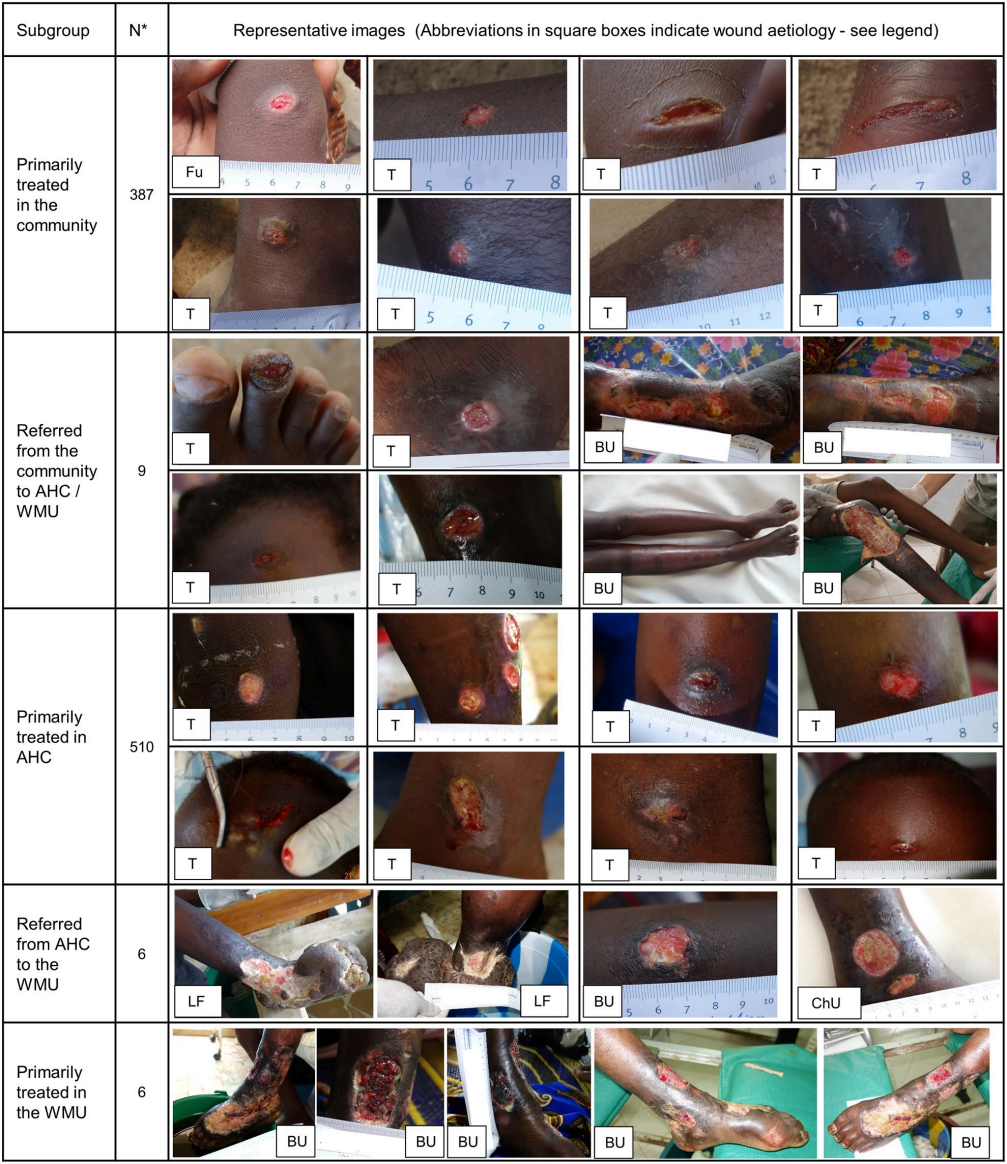
Representative images of the wounds treated within the study stratified by health service level. In the large subgroups “Primarily treated in the community” (N = 387) and “Primarily treated in AHC” (N = 510) the images were randomly selected to rule out selection bias. Multiple pictures are shown for patients with complicated wounds. Abbreviations in the square boxes indicate the wound aetiology: Fu = furuncle (ulcerated), T = Mechanical trauma, BU = Buruli Ulcer, LF = Late-stage lymphatic filariasis, ChU = Chronic wound of unknown origin. AHC: Ahondo Health Centre; WMU: Wound Management Unit. * = Number of wounds.

A total of 923 wounds were included in the study. Thirty-nine patients with 49 wounds were lost to follow-up, 2 wounds belonged to a patient who deceased before follow-up completion. The dataset was finalized on March 4^th^, 2021, for long-term observation of complicated chronic wounds. Median follow-up time was 35 days (IQR 17–98). Each patient had on average 1.2 wound events in a total of 698 wound events. The most frequent aetiology was mechanical trauma, followed by furuncles, burns and BU (Table 1). Most wounds were acute (70.7%, 595/841) and smaller than 5 cm^2^ in size (87.2%, 464/532). At enrolment, 14.9% (119/799), 4.1% (33/799) and 3.0% (24/799) of wounds showed signs of local, deep, and systemic secondary bacterial infection, respectively. Fig 3 shows a representative range of wounds stratified by health service where treatment was provided.

**Table 1:** Study endpoints for wounds stratified by main aetiology (confirmed diagnosis).

Aetiology			Wound closure		Time to wound closure		Duration of treatment		Sec. bact. infect. at enrolment	
			Den	N (%)	Den	Median (Mean) in days	Den	Median (Mean) in days	Den	N (%)
**Mechanical trauma**	Total N (%)	766 (84.8)	733	733 (100)	145	11.0 (17.6)	731	6.0 (13.9)	676	122 (18.0)
	LFU N (%)	33 (68.8)								
Requiring surgical treatment	Total N (%)	16 (1.8)	16	16 (100)	4	18.0 (20.3)	16	14.5 (21.6)	15	1 (6.7)
	LFU N (%)	0 (0)								
Treated with local wound care alone	Total N (%)	750 (83.1)	717	717 (100)	141	11.0 (17.5)	715	6.0 (13.7)	661	121 (18.3)
	LFU N (%)	33 (68.8)								
**Furuncles**	Total N (%)	41 (4.5)	35	35 (100)	5	13.0 (22.2)	35	5.0 (11.3)	37	20 (54.1)
	LFU N (%)	6 (12.5)								
**Burns**	Total N (%)	32 (3.5)	30	30 (100)	5	21.0 (20.2)	30	1.0 (5.2)	30	9 (30.0)
	LFU N (%)	2 (4.2)								
**Buruli ulcer**	Total N (%)	30 (3.3)	27	25 (92.6)	15	107.0 (134.7)	25	104.0 (152.1)	21	13 (61.9)
	LFU N (%)	3 (6.3)								
**Yaws**	Total N (%)	14 (1.6)	14	14 (100)	2	-	14	10.5 (24.6)	6	0 (0)
	LFU N (%)	0 (0)								
**Chronic wounds of unknown aetiology**	Total N (%)	8 (0.9)	4	2 (50)	0	-	2	-	7	5 (71.4)
	LFU N (%)	4 (8.3)								
**Others**	Total N (%)	12 (1.3)	12	12 (100)	6	-	12	-	8	5 (62.5)
	LFU N (%)	0 (0)								
**Subtotal**	Total N (%)	903 (100)	855	851 (99.5)	178	-	849	-	785	174 (22.2)
	LFU N (%)	48 (100)								
**Missing data on aetiology**	Total N	20	19	19 (100)	2	-	19	-	14	2 (14.3)
	LFU N	1								
**Total**	Total N	923	874	870 (99.5)	180	-	868	-	799	176 (22.0)
	LFU N	49								

A total of 903 (903/923, 97.8%) wounds were considered for analysis, 20 wounds were excluded because of missing data on aetiology. Analysis on wound closure was performed on wounds with completed follow-up; analysis on time to wound closure was performed on wounds with a gap of 7 days or less between the last wound dressing change and documented wound closure; analysis on duration of treatment was performed on wounds with documented wound closure and date of last follow-up; analysis on secondary bacterial infection at enrolment was performed on wounds where all infection criteria were assessed and documented at enrolment. LFU = Lost to follow-up; Den = Denominator used for percentage calculation in each subgroup. Secondary bacterial infections include local, deep, and systemic infections reported at enrolment. No calculations were performed for subgroups with small numbers or the heterogenous subgroup “Others”, the corresponding cells have been greyed out.

### Outcomes

#### Proportion of wounds closed

Nearly all wounds (94.7%, 874/923) completed follow-up and were included in the calculation of the proportion of wounds that closed (99.5%, 870/874). Among wounds with a confirmed diagnosis 99.5% (847/851) closed without a revision of the initial presumptive diagnosis, 0.5% (4/851) required one revision, and no wound required two or more revisions. All wounds that completed treatment in the community and at AHC closed, compared to 76.9% (10/13) of the wounds that were referred to a higher health service level and 83.3% (5/6) of the wounds primarily treated in the WMU (Table 2). Of the four wounds that did not close by the end of the study period, two wounds were chronic wounds of unknown aetiology, two wounds were due to BU (Table 1, for details see S1 Notes—section B).

**Table 2:** Study endpoints for wounds stratified by health service level.

Health service level			Wound closure		Time to wound closure		Duration of treatment		Secondary bacterial infections at enrolment	
			Den	N (%)	Den	Median (Mean) in days	Den	Median (Mean) in days	Den	N (%)
**Primarily treated in community**	Total N (%)	387 (42.9)	369	368 (99.7)	76	10.0 (16.2)	366	6.5 (13.2)	350	36 (10.3)
	LFU N (%)	18 (36.7)								
Completed in community	Total N (%)	378 (41.9)	362	362 (100)	75	10.0 (15.8)	360	6.0 (13.1)	342	33 (9.6)
	LFU N (%)	16 (32.7)								
Referred from community to AHC or WMU	Total N (%)	9 (1.0)	7	6 (85.7)	1	-	6	14.5 (18.2)	8	3 (37.5)
	LFU N (%)	2 (4.1)								
**Primarily treated in AHC**	Total N (%)	510 (56.5)	479	477 (99.6)	98	16.0 (31.4)	477	6.0 (20.4)	429	133 (31.0)
	LFU N (%)	31 (63.3)								
Completed in AHC	Total N (%)	504 (55.8)	473	473 (100)	98	16.0 (31.4)	473	6.0 (19.2)	427	132 (30.9)
	LFU N (%)	31 (63.3)								
Referred from AHC to WMU	Total N (%)	6 (0.7)	6	4 (66.7)	0	-	4	194.0 (169.3)	2	1 (50.0)
	LFU N (%)	0 (0)								
**Primarily treated in WMU**	Total N (%)	6 (0.7)	6	5 (83.3)	4	170.0 (160.5)	5	196.0 (230.0)	5	5 (100)
	LFU N (%)	0 (0)								
**Subtotal**	Total N (%)	903 (100)	854	850 (99.5)	178	-	848	-	784	174 (22.2)
	LFU N (%)	49 (100)								
**Missing data on health service level**	Total N	20	20	20 (100)	2	-	20	-	15	2 (13.3)
	LFU N	0								
**Total**	Total N	923	874	870 (99.5)	180	-	868	-	799	176 (22.0)
	LFU N	49								

A total of 903 (903/923, 97.8%) wounds were considered for the analysis, 20 wounds were excluded because of missing data on health service of treatment. Analysis on wound closure was performed on wounds with completed follow-up; analysis on time to wound closure was performed on wounds with a gap of 7 days or less between the last wound dressing change and documented wound closure; analysis on duration of treatment was performed on wounds with documented wound closure and date of last follow-up; analysis on secondary bacterial infection at enrolment was performed on wounds where all infection criteria were assessed and documented at enrolment. LFU = Lost to follow-up; Den = Denominator used for percentage calculation in each subgroup. Secondary bacterial infections include local, deep, and systemic infections reported at enrolment or during follow-up. Subtotals which have not been used for analysis and subgroups with small numbers have been greyed out.

### Time to wound closure and duration of treatment

Analysis of time to wound closure was performed on 180 wounds (20.7%, 180/870) for which closure was documented within 7 days after the last wound dressing. Wounds primarily treated in the community, AHC and the WMU closed within a median time of 10, 16 and 170 days, respectively (Table 2). Time to closure was longer for chronic wounds than for acute wounds (median of 72 and 13 days, respectively) and for aetiologies requiring specific treatment, such as BU (107 days–see Table 1). Wounds with secondary bacterial infection at enrolment had longer time to closure than non-infected wounds (median of 34.5 and 10 days, respectively). Similar results were observed for duration of treatment, with longer duration for chronic compared to acute wounds (17.5 and 5.0 days, respectively), for aetiologies requiring specific treatment (Table 1) and for infected compared to non-infected wounds (11.5 and 5.0, respectively). Among traumatic wounds, burns and furuncles smaller than 5 cm^2^ at enrolment, 72.0% (59/82) closed in less than 20 days (mean and median of 2.7 and 2 wound care visits, respectively) and 71.1% (290/408) required less than 2 weeks of treatment duration (mean and median of 2.1 and 2 wound care visits, respectively).

#### Time-dependent wound size analysis

Of the wounds with full assessment at the Health Centre / WMU, 12.8% (68/532) were larger than 5cm^2^ at enrolment. We selected 16 wounds with a full set of size measurements for assessing the Flanagan criteria at 3 weeks after initiation of treatment and < 30% wound area reduction to predict healing at week 12. Prediction was accurate in 14/16 wounds (87.5%).

#### Secondary bacterial infections

At enrolment, 22.0% (176/799) of wounds presented evidence of secondary bacterial infection, mostly local infection. Wounds treated in the community, AHC and WMU presented with secondary bacterial infections at enrolment in 10.3% (36/350), 31.0% (133/429) and 100% (5/5) of cases, respectively; at follow-up in 62.5% (5/8) of wounds referred from the community to AHC and in all wounds (6/6) referred from AHC to the WMU, compared to 7.8% and 37.6% of wounds completing treatment in the community and AHC, respectively. Compared to acute wounds chronic wounds had higher proportions of secondary bacterial infections at enrolment (21.3% vs 39.5%) and at follow-up (23.8% vs 46.3%).

#### Adherence to treatment (recaptured patients and loss to follow-up)

Most wounds (68.3%, 630/923) required at least one, 18.2% (168/923) multiple recaptures. The proportion of wounds in need of recapturing was 60.7% (235/387), 74.1% (378/510) and 16.7% (1/6) among patients primarily treated in the community, AHC and the WMU, respectively. Most wounds primarily treated in the community and in AHC had already closed at the first successful recapture, 68.4% (162/237) and 67.0% (246/367), respectively. Highest proportion of recaptures were seen for wounds due to mechanical trauma that had required surgical treatment (93.8%, 15/16) and chronic wounds of unknown origin (87.5%, 7/8). Among the former, 13/15 had closed at time of first successful recapture and 2/7 among the latter. BU had the lowest proportion of recaptures (53.3%, 16/30). Highest proportions of loss to follow-up were observed among patients with chronic wounds of unknown origin (3/6 –for details see S1 Notes—section C), furuncles (4/22) and BU (2/20—for details see S1 Notes—section D). Most frequent causes of loss to follow-up despite a recapturing attempt were non-adherence to treatment recommendations (14/39) and emigration outside the Ahondo Health Area (13/39).

#### Wound-related pain

Most wounds (76.8% 624/813) were not described as painful at enrolment. Wound-related pain was reported more frequently in wounds with local infection (22.6%, 24/106), deep infection (43.8%, 14/32) and systemic infection (54.2%, 13/24) than in non-infected wounds (19.4%, 106/545). At enrolment 35.7% (10/28) of BU-related wounds were reported as painful.

#### Local wound care

Data on local wound care was available for 92.4% of follow-ups (3584/3877). Local wound care of wounds that completed treatment in AHC comprised mostly wound dressings with sterile gauzes either dry (57.4%) or soaked in a Povidone iodine solution (31.0%) for short courses of treatment in case of secondary bacterial infection. Community health workers mostly used dry gauzes (87.4%) for local wound care.

#### Specific and additional treatment

Overall, 20.3% (114/561) of patients received additional treatment such as antibiotics, analgesics, suturing, and anti-tetanus immunoglobulins. All patients referred to the WMU required antibiotics and/or surgical interventions, such as debridement or split skin grafting, and received nutritional support. One patient required amputation of the lower leg; two patients received split skin grafting. All BU and yaws cases were specifically treated following WHO/national guidelines by the national control program.

## Discussion

The development of our wound management model “Identify and treat early” is a response to the neglect of wounds in rural areas of sub-Saharan Africa [6,19]. Little is known about the burden, and wound care at the community level is largely lacking. Wounds in rural areas are related to living, working and mobility conditions in addition to region-specific causes (skin NTDs) [5,8,20–22]. Limited access to and lacking standards of wound care including hygiene deficits lead to wound complications and chronic wounds [5]. “Identify and treat early” targets the problem upstream, at the household level, to reduce suffering, cost and to cope with health services access problems downstream at health centres and district hospitals. In contrast to vertical control programmes and surveys targeting specific diseases associated with skin lesions, such as leprosy, BU and yaws, our model addresses all wounds independent of the cause [8,23,24].

To our knowledge, this is the first study in rural Sub-Saharan Africa with a horizontal approach covering all skin wounds independent of the cause. It is in line with the WHO-recommended integrated strategy for skin NTDs [8] and extends the concept to non-NTD aetiologies. Similar approaches to skin NTDs and common skin diseases have provided promising results [23,25,26].

To overcome the inherent selection bias of school-, health services-based and disease-specific surveys we conducted our study in a population under HDSS surveillance [24] and combined a household-based (active case finding) with a health services-based survey (passive case finding) [13].

The lack of a simple, generally useful, and accepted classification for wounds is a problem, both for documenting wounds and for healthcare personnel to triage wounds to the appropriate health care level. Aetiological factors come into play (e.g. region specific causes such as Buruli Ulcer and yaws; the nature of the injury), as well as chronicity, area and depth of wounds (superficial or deep) and wound complications (most importantly, wound infection with a severity range from local infection to systemic manifestations and chronic wound development).

We trained the local healthcare personnel to recognize wounds that require specific treatment (BU, yaws, tuberculosis), to distinguish between superficial (‘minor wounds’, such as skin abrasions) and deep wounds, and to classify secondary bacterial infections into local, deep, and systemic infections, to understand and apply the principles of hygiene and to recognize indications for wound debridement and skin grafting. Training of health staff and wound management were based on resources and materials available in the national health care system and on WHO recommendations [1,7] to increase the likelihood of sustainability after research is over. The healthcare personnel also learned to triage wounds into those that can be treated on their respective levels (community, health centre, district hospital) and those that require referral to higher levels. Training also included recognition of red flags during treatment signifying failure of wound management and / or complications requiring referral. The decision process was aided by supervision of the study team, both on site and through smartphone communication.

In countries with limited resources wound diagnosis cannot rely on sophisticated laboratory diagnostics. In rural areas this is aggravated by the distance to health care facilities and cost. We resorted to good clinical practice of presumptive diagnosis refined over time by repeated careful follow-up of patients. This strategy proved to be effective, only 4 presumptive diagnoses (0.5%) had to be revised during follow-up, because of treatment failure or upon review by clinical investigators.

Across all three health service levels–community, health centre, district hospital–, 99.5% (870/874) of all wounds enrolled between May 2^nd^, 2019, and March 4^th^, 2020 had closed at the final assessment on March 4^th^, 2021. At the end of the study period 42.9%, 56.5% and 0.7% of all wounds were primarily treated at the community, health centre and district hospital level and closed with a median time of 10, 16 and 170 days, respectively. Very few (1.7%) of wounds required referral to higher levels of the health services after initial treatment, supporting the triage procedures adopted by the wound management model as discussed above. The distribution of the frequency of secondary bacterial infections across the three health care levels– 10.3%, 31.0% and 100% of wounds infected in the community, health centre and district hospital, respectively–points into the same direction that more complicated wounds were managed at higher, appropriate levels.

Based on these results, we pragmatically classified wounds by health service level where they had been treated. Fig 3 illustrates the consistency of this approach. In the continuing exploration and adaptation of the wound management model, we will refine the criteria for selecting the appropriate health service level for the management of wounds in rural communities and will incorporate them into the training program of healthcare personnel.

The fact that more than 40% of all wounds enrolled in the study could be treated in the community by healthcare workers and closed within a median time of 10 days should have a significant impact on the reduction of chronic wound development. We will quantify this impact in the ongoing wound management study combined with repeated household-based wound surveys within the Taabo HDSS.

Time to wound closure increased, as expected, from the community to the health centre and the district hospital with 10, 16 and 170 days, respectively. Wounds with uncertain dates of closure due to delayed assessment at recapture were excluded from the analysis of time to closure. For the interesting pragmatic outcome–the duration of treatment sufficient for wound closure–the recaptured patients with healed wounds could instead be included in the analysis with the calculation of the time up to the last wound care follow up. Similar to time to closure, results showed an increasing duration of treatment from lower to higher levels of health services, again demonstrating the benefits of identifying and treating wounds early.

The impact on suffering and cost of late identification and treatment was obvious in two severe BU cases and in a patient with end-stage lymphatic filariasis, requiring skin grafting and amputation, respectively (see Fig 3). As expected, BU required much longer treatment time until wounds closed compared to other aetiologies, but the BUs of all our patients adhering to treatment recommendations closed (25/27) or showed evidence of healing under treatment at end of the study period. Overall, follow-up of BU patients has been satisfactory, except for two patients, a woman with longstanding inactive BU who postponed treatment due to social obligations and a woman with inactive BU who emigrated out of the country. The impact of early identification and treatment could also be shown when comparing time to wound closure of acute and chronic wounds with 13 vs 72 days.

One of the main driving forces from acute to chronic wounds were repeated, often persisting secondary bacterial infection. This leads in an unknown proportion of patients to catastrophic events of systemic infection and sepsis and in most patients to an increase of wound size and depth, arrested wound healing (if surgical intervention such as wound debridement is not performed) and local sequelae (such as contractures in wounds anatomically related to joints).

One of the major drawbacks in wound management is to miss the point in time when wounds become arrested, and closure is inhibited by an increasingly unresponsive wound edge and base. We could show that monitoring of wound area reduction with Flanagan’s criteria [14] facilitates recognition of wounds in need of refinement or revision of management. Prediction was accurate in 87.5% of wounds with an initial size greater than 5 cm^2^.

We assessed the patient adherence to treatment. Most wounds (68.3%) required at least one recapture due to interruption of follow-up. We made any effort to recapture patients, help them to accomplish wound closure and to get an insight into the reasons of non-adherence to treatment. It was very reassuring to find that most wounds (68.1%) had already closed at time of recapturing, a simple and understandable reason for not attending scheduled follow-ups. Few (5.3%) wounds were lost to follow-up despite our recapturing efforts. A third of patients lost to follow-up were reported to have migrated outside the study area, as expected in a rural area with many seasonal workers and high mobility between villages. Nonetheless, the high proportion of recapturing required and the loss to follow-up of patients due to non-adherence to treatment recommendations needs attention to identify underlying socio-cultural and economic factors and to adapt the model to the needs of the local population.

Our study has several limitations. The study enhanced the supply of materials such as dressing materials, local disinfectants, and antibiotics, to bridge local shortage and to support patients who could not afford to pay by themselves, which is a substantial proportion of patients in poor rural communities. Hospitalized patients also received nutritional support. A further study-related input was the assessment of basic infrastructural requirements and the refurbishment of wound showers, drinking quality water, and waste disposal. This input was, however, adapted to the community setting, of low cost and provided multiple benefits to the health services other than for wound care. We discussed and decided each step with the local community to achieve long-term sustainability. We did not include cost assessment into the baseline study. It would have overburdened the project and our capacity, and there would have been a very high risk of bias, as during the implementation phase project inputs are naturally high. Costs will only come down to acceptable levels through negotiating with the health authorities the best fit of the model within the existing health care system, including the functioning of the material supply chain and the integration of the maintenance of the infrastructure development and of wound management training into the health services.

Also, the mental health impact of chronic, complicated, and stigmatizing (BU) wounds [2–4] has not been systematically assessed in the baseline study.

In summary, the community-centred wound management model based on early identification and treatment of all wounds independent of the aetiology has great potential to save cost and healthcare resources in addition to the reduction of suffering. The study shows that with adherence to WHO recommendations, basic infrastructure for wound management in place and training, most wounds can be treated by local healthcare personnel, close to the community and with a high success rate. Flanagan’s criteria are a simple method that can be used in settings with limited resources to monitor progress of wound healing and to revise the presumptive diagnosis and wound management. Recognition of and a well-functioning referral system are essential for complicated and specific wounds (leprosy, BU, yaws) in need of more intensive treatment at higher health service levels. A community-centred and horizontal approach to skin wounds found high acceptance by the local population contributing to the success of the wound management model. In the currently ongoing second project phase of three years, we carry out assessments of the impact of the wound management strategy on the burden, clinical epidemiology, cost and mental health issues of wounds. The results will have major implications for the sustainability and scalability of the model.

## Supporting information

S1 Notes Notes on selected patients. (DOCX)

S1 STROBE Checklist STROBE Statement—Checklist of items that should be included in reports of observational studies. (DOCX)
