# Supplementary material for: Community-based wound management in a rural setting of Côte d’Ivoire
Source: PLoS Negl Trop Dis. 2022 Oct 13;16(10):e0010730. doi: 10.1371/journal.pntd.0010730 (PMC9560516; doi:10.1371/journal.pntd.0010730)
Supplement: S1 Notes — (DOCX) [file pntd.0010730.s001.docx]

# Notes on selected patients

## Patient deceased during the study period

64-year-old male patient with a history of hypertension presenting with second degree burns of the last phalanx of two fingers of the right hand that he had procured himself two weeks prior. At the physical exam he had a heart rate of 89 beats per minute and was afebrile (37.2⁰C). Both fingers presented no signs of deep secondary bacterial infection, particularly no enlarged regional lymph nodes. He was treated with amoxicillin/clavulanic acid and metronidazole. He did not present for follow-up treatment and died three days afterwards in unclear circumstances.

## Wounds that did not close by end of the observation period

Wounds 1 and 2 were classified as chronic wounds of unknown origin and belonged to a 20-year-old female patient. Both wounds were located at the left ankle and had a wound size of 6 cm^2^ and 3 cm^2^, respectively. Follow-up was interrupted multiple times by the patient, who resorted several times to traditional treatment. Both wounds showed evidence of recurrent secondary bacterial infections after follow-up interruptions. The patient finally accepted to be hospitalized at the wound management unit (WMU) of Taabo district hospital. No specific aetiology could be identified, but both wounds showed reduction in size under basic wound management, without specific treatment. As of December 17^th^, 2021, one wound has closed, one wound had a size of 4.5 cm^2^ and showed evidence of re-epithelialization.

Wound 3 belonged to a 44-year-old male patient with Category III BU of the left lower leg and foot. He was hospitalized at the WMU of Taabo district hospital with multiple wounds and after BU-specific treatment (Rifampicin and Clarithromycin) and several months of intensive wound management received skin grafting. At the end of the observation period five of six wounds had closed, as of December 17^th^, 2021, one wound remained open with a wound surface of 2 cm^2^ and showed evidence of re-epithelialization.

Wound 4 belonged to a 10-year-old female patient with Category III BU of the right knee and lower leg. She was hospitalized at the WMU of Taabo district hospital and after BU-specific treatment (Rifampicin and Clarithromycin) and several months of intensive wound management received skin grafting. As of December 17^th^, 2021, a small residual wound with a wound surface of 2 cm^2^ remained open at the border of the skin graft site and showed evidence of re-epithelialization.

## Loss to follow-up among chronic wounds of unknown origin

Patient 1 had two wounds most likely due to chronic osteomyelitis following osteosynthesis of the lower leg performed years ago. He was recaptured repeatedly but stopped attending appointments. Patient 2 had a chronic wound most likely traumatic in origin, was recaptured twice, but stopped attending appointments. Patient 3 had a chronic wound at the ankle, did not attend health services for treatment, was recaptured, but did not attend the planned appointment.

## Loss to follow-up among BU-related wounds

Patient 1 had inactive BU with a small-sized wound of the forearm surrounded by scar tissue. She attended multiple follow-ups, was recaptured repeatedly after multiple interruptions, but finally moved outside the country. Patient 2 had Category III BU of the lower leg, comprising two wounds, with beginning lymphedema. She reported to have completed BU-specific antibiotic treatment. Despite multiple recaptures she did not attend the health services for treatment and refused to be hospitalized at the WMU of Taabo district hospital for intensive treatment.
